# Toward Non-Invasive Neurological Biomarker Monitoring: Dopamine Sensing in Tears with Laser-Induced Graphene Electrochemical Sensors

**DOI:** 10.1021/acsomega.6c03287

**Published:** 2026-06-09

**Authors:** Lucas Minghini Gonçalves, Bruno Vasconcellos Lopes, Bruno da Silveira Noremberg, Raphael Dorneles Caldeira Balboni, Guilherme Kurz Maron, Anderson Thesing, Daiane Dias, Irene Teresinha Santos Garcia, Sabir Khan, Neftali Lenin Villarreal Carreno

**Affiliations:** † Graduate Program in Materials Science and Engineering, Technology Development Center, Federal University of Pelotas, 96010-000 Pelotas, Rio Grande do Sul, Brazil; ‡ Center for Embedded Devices and Research in Digital Agriculture (CEDRA), São Leopoldo, RS 93025-753, Brazil; § Northern Regional Technological Institute (ITR Norte), Technological University of Uruguay (UTEC), 40000 Rivera/Rivera, Uruguay; ∥ Institute of Physics, 28124 Universidade Federal do Rio Grande do Sul, Porto Alegre RS 91501-970, Brazil; ⊥ Universidade Federal do Rio Grande (FURG) − School of Chemistry and Food, Av. Itália S/N, km. 8 (Carreiros), Rio Grande, RS 96203-900, Brazil; # Federal University of Rio Grande do Sul, Department of Physical Chemistry, Porto Alegre, RS 91501-970, Brazil

## Abstract

Dopamine plays a crucial role in motor control, cognition, and emotional regulation, and its abnormal levels are associated with disorders such as Parkinson’s disease and schizophrenia, highlighting the need for sensitive, selective, and noninvasive detection methods. This study reports the development of a high-performance, nonenzymatic electrochemical sensor based on laser-induced graphene, functionalized with nickel nitrate and urea, for the detection of dopamine. Cyclic voltammetry and differential pulse voltammetry were employed to assess the sensor’s selectivity and overall performance. In-depth characterization by scanning electron microscopy and Raman spectroscopy confirmed the successful formation of a porous and electroactive graphene structure, uniformly functionalized with nickel ions and nitrogen-containing groups. These modifications enhanced electron transfer rates and increased the number of active sites for dopamine oxidation. Electrochemical measurements demonstrated excellent performance, with a linear detection range of 0.25–16.44 μmol·L^–1^, a limit of detection of 17.86 nmol·L^–1^, and a limit of quantification of 54.14 nmol·L^–1^, with *R* ^2^ = 0.98 in phosphate-buffered solution. In synthetic tear fluid, the sensor maintained a reliable response across four different concentrations ranging from 3.23 to 9.32 μmol·L^–1^. Furthermore, the sensor exhibited excellent analytical performance in real matrices, achieving recovery rates close to 100% in real sample analyses.

## Graphical abstract

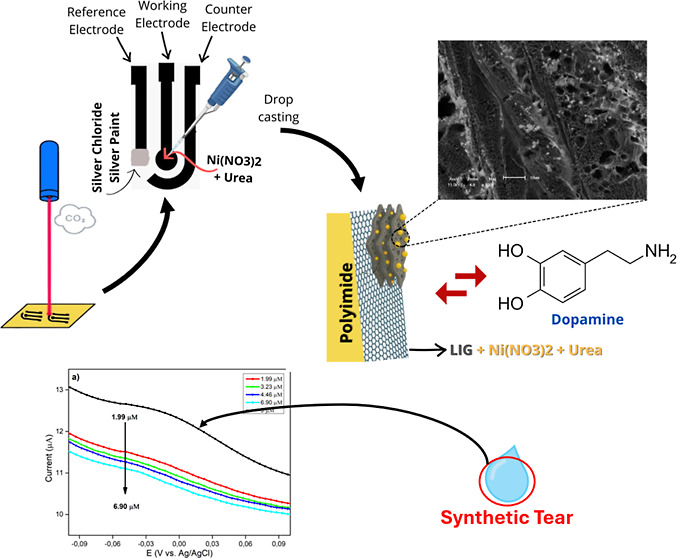

## 1 Introduction

Dopamine (DA) is a catecholaminergic neurotransmitter essential to the central nervous system, playing a critical role in modulating cognitive, motor, and reward processes, acting as a mediator of the sensation of well-being.^1^ Epidemiological studies have shown that alterations in DA levels are associated with neurodegenerative and psychiatric disorders, including Parkinson’s disease, schizophrenia, Alzheimer’s disease, and depression.^2,3^ These conditions are directly related to dysfunctions in dopaminergic systems.^4^

For healthy individuals, plasma DA concentrations range between 0 and 30 pg·mL^–1^ (195.8 pmol·L^–1^),^5^ while in tear fluid measured via the Schirmer technique, reported levels range from 152 to 519.1 pg·mL^–1^ (3.38 μmol·L^–1^).^6,7^ Currently, DA monitoring is predominantly based on invasive methods such as urine analysis, blood sampling, or brain implants. While effective, these approaches present limitations regarding practicality, patient risk, and discomfort.^8,9^

As a promising and yet noncommercialized alternative, electrochemical sensors have emerged as a promising alternative for the detection of DA in biological fluids due to their high sensitivity, selectivity, and possibility of miniaturization.^10,11^ These devices convert chemical interactions between the analyte and the electrode into measurable electrical signals, offering advantages such as low cost,^12,13^ portability,^14^ and the ability for *in situ* analysis. The chemical composition of these sensors plays a fundamental role in their performance and applicability, often relying on conductive materials, semiconductors, or nanocomposites to enhance sensitivity and selectivity.^15^ The electrochemical performance of sensors is intrinsically associated with the architecture of the transducer material.^16,17^

In this context, carbon-based materials—especially graphene—have emerged as ideal platforms due to their high conductivity, large surface area, and chemical stability.^18^ Laser-induced graphene (LIG) obtained via laser carbonization of polymeric substrates represents a significant advancement in this field. LIG exhibits a three-dimensional porous structure, tunable electrocatalytic properties, and compatibility with scalable manufacturing processes.^19^ Due to its ease of fabrication and low cost, LIG has been widely explored for application in wearable devices, environmental monitoring, and health.^20–22^ Urea was employed within the LIG framework to enhance pore enlargement, thereby facilitating the deposition of nickel particles.^23,24^ In parallel, nickel-based compounds have attracted interest in sensor development due to their redox activity, low cost, and electrocatalytic efficiency.^25^ Moreover, functionalizing the LIG surface with nickel can enhance sensitivity and selectivity toward specific molecules such as DA through an electronic coordination mechanism.^26^

In this study, we report on the development of a low-cost, nonenzymatic electrochemical sensor based on LIG functionalized with nickel nitrate and urea for the detection of DA under standard laboratory conditions and synthetic tear fluid. Cyclic voltammetry (CV) and differential pulse voltammetry (DPV) were employed to evaluate the sensor’s electroanalytical performance, with a focus on clinical diagnostics and noninvasive monitoring applications.

## 2 Experimental Section

### 2.1 Materials

Sodium dihydrogen phosphate and disodium hydrogen phosphate were supplied by Synth (São Paulo, Brazil). Nickel­(II) nitrate hexahydrate, urea, dopamine hydrochloride, l-lactate, β-d-glucose, l-ascorbate, and bovine serum albumin were supplied by Sigma-Aldrich (São Paulo, Brazil). All chemicals used in the experiments were analytical grade. Ultrapure water (25 °C), obtained from a Milli Q Direct-0.3 purifier (Millipore), was used for both the preparation of the solutions and the synthesis of the materials.

### 2.2 Electrode Fabrication and Functionalization

Three-electrode platforms were developed for sensor fabrication, comprising a working, counter, and reference electrodes. Electrode layouts were designed in Inkscape vector graphics software. The electrodes were fabricated via a two-step carbonization process using direct laser writing (DLW) on polyimide (Kapton) film. A CO_2_ laser (10.2 μm) engraving machine (Visutec Router VS3020P) was employed, operating at a scan speed of 100 mm·s^–1^ and a power output of 3.2 W. In the first step, the laser was used to engrave the Kapton film, creating a complete three-electrode design. For the preparation of the structure-modifying solution for LIG, 0.1 mol·L^–1^ nickel nitrate and 0.3 mol·L^–1^ urea were dissolved in ultrapure water. The working electrode was then modified via drop-casting with 20 μL of an aqueous solution of 0.1 mol·L^–1^ nickel nitrate and 0.3 mol·L^–1^ urea. After allowing it to dry for 4 h, a second DLW was applied to further process the modified layer. Different functionalization was tested, including the combinations of LIG/urea, LIG/Ni­(NO_3_)_2_, and LIG/Ni­(NO_3_)_2_ + Urea. To determine the optimal urea concentration, sensors were also tested with concentrations ranging from 0.1 to 0.4 mol·L^–1^. A layer of commercial Ag/AgCl conductive ink (ALS da BAS Inc., Tokyo, Japan) was applied to the pseudoreference electrode. Conductive silver ink was applied to all three electrodes to improve electrical contact. Finally, a hot-glue barrier was added around the electrode area to create a containment well for the electrolyte solution to be deposited during electrochemical measurements. Figure 1 illustrates the methodology used for electrode fabrication.

**Figure 1:**
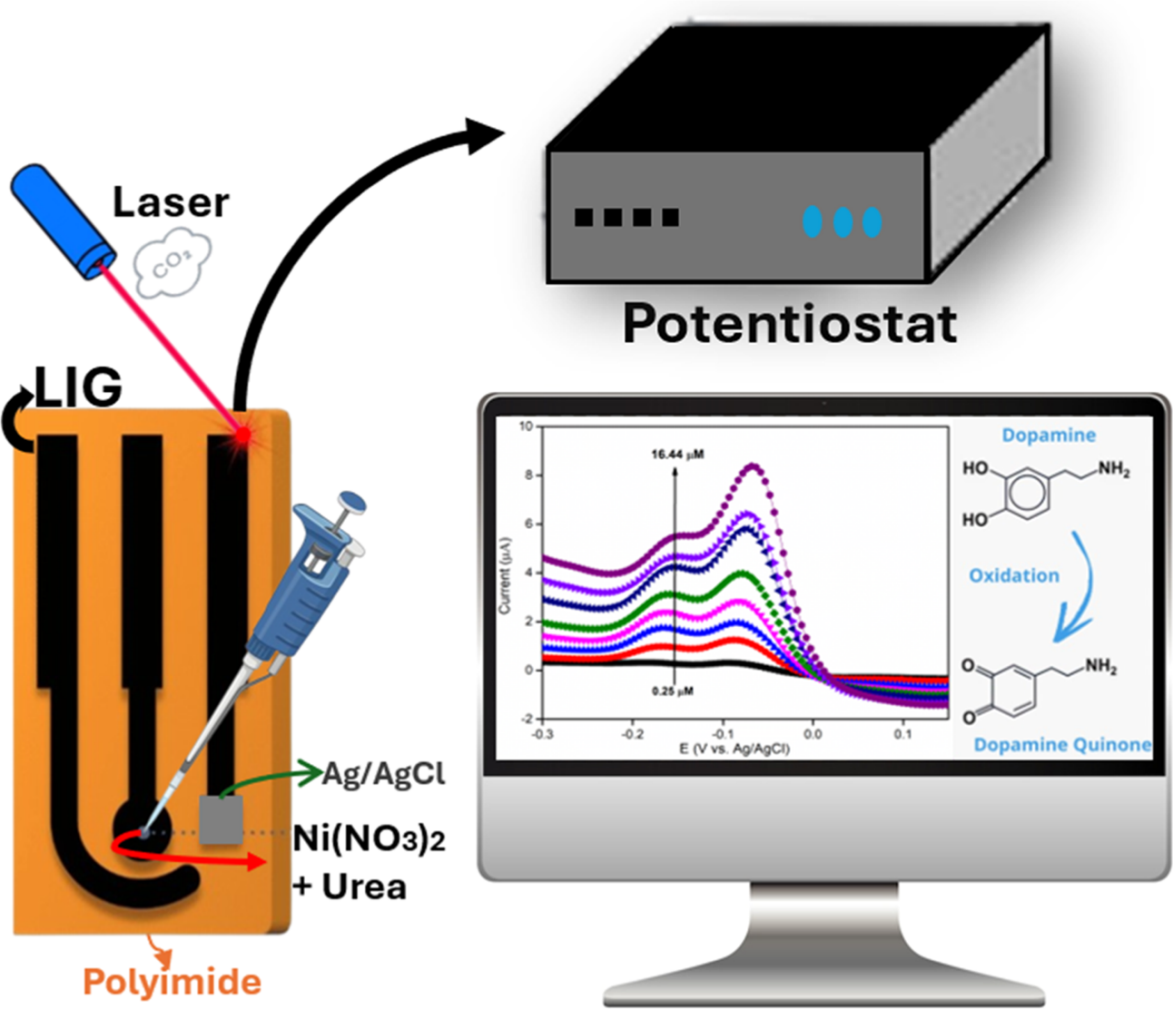
Schematic representation of the LIG fabrication process, leading to the results with dimensions of 10 × 20 mm.

### 2.3 Apparatus

Raman spectroscopy was performed using a WITec alpha300 confocal Raman microscope equipped with a 50× objective and a 532 nm excitation laser. All samples were analyzed under identical experimental conditions, with an integration time of 1 s and 60 accumulated scans, and with laser power maintained constant across measurements. For each electrode, spectra were collected from three different regions and averaged to ensure a representative sampling. The acquired spectra underwent sequential processing, including baseline correction through polynomial fitting, normalization, and Gaussian smoothing with a standard deviation of 10. Spectral analysis focused on integrated peak areas rather than relative intensities to ensure an accurate quantitative comparison. Control measurements conducted on replicate samples prepared under identical conditions confirmed the reproducibility of the spectra with no observable significant peak shifts between measurements. This rigorous analytical approach ensured the reliable characterization of carbonaceous materials while minimizing measurement variability. The consistent experimental parameters across all samples enabled a direct comparison of spectral features between different material compositions. Scanning electron microscopy (SEM) was performed using a Shimadzu SSX-550, at magnifications of 100×, 500×, and 1500×, to investigate LIG morphology. All electrochemical tests were conducted on a Metrohm Autolab PGSTAT302N potentiostat/galvanostat.

### 2.4 Preparation of Solutions and Real Samples

For the blank sensor samples, a 0.1 mol·L^–1^ PBS solution was used, prepared using NaH_2_PO_4_·H_2_O and Na_2_HPO_4_ in distilled water, followed by 10 min of sonication. The analytical solution consisted of 1 mmol·L^–1^ DA, prepared by dissolving dopamine hydrochloride in 0.1 mol·L^–1^ PBS and sonicating it for 10 min. The synthetic tear solution was prepared following the protocol by Reid (2015)^27^ and included 150 mmol·L^–1^ PBS, 3 mmol·L^–1^ l-lactate, 0.05 mmol·L^–1^ β-d-glucose, 0.18 mmol·L^–1^ l-ascorbate, 5.4 mmol·L^–1^ urea, and 0.2 mg·mL^–1^ BSA. For performance evaluation, triplicate measurements were performed using CV and DPV. An interference study was conducted, where each synthetic tear component was individually mixed with the 1 mmol·L^–1^ DA solution to analyze the behavior of the LIG/Ni­(NO_3_)_2_ + Urea sensor, as shown in Figure S4.

### 2.5 Electrochemical Measurements

Electrochemical experiments were used to evaluate the sensor’s working range, DA detection capability, and the corresponding current responses. CV measurements were conducted in the potential window from −0.5 to 0.5 V at a scan rate of 0.05 V·s^–1^. DPV tests were performed from −0.35 to 0.35 V, with a step potential of 0.005 V, and a scan rate of 0.05 V·s^–1^. Before each test, the sensor was activated by applying five CV cycles in 400 μL of 0.1 mol·L^–1^ PBS. A baseline analysis was then recorded and used as a reference to calculate current differences in DA presence. The PBS was then replaced with 400 μL of 100 μmol·L^–1^ dopamine solution. Analytical curves were constructed based on oxidation peak currents from DPV triplicates. Mean and standard deviation values were calculated. DA concentrations ranged from 0.25 to 16.44 μmol·L^–1^ in PBS, and from 1.99 to 6.90 μmol·L^–1^ in synthetic tears.

## 3 Results and Discussion

### 3.1 Physical-Chemical Characterization of the Electrodes

During laser irradiation of the polyimide substrate surface, different structural configurations of LIG can be obtained. These configurations vary according to the laser beam’s incidence parameters and the irradiation pattern (linear or dot).^28^ The LIG exhibits a highly interconnected porous structure composed of three-dimensional graphene nanosheets, which are considered ideal for electrochemical sensor electrodes.^19,29^ The SEM images were used to analyze the matrix structure of LIG, LIG/Urea, and LIG/Ni­(NO_3_)_2_ + Urea. The analysis revealed that the LIG’s structural behavior was modified, with pore growth and particle deposition within the porous structures. Figure 2a–c) shows the SEM analysis of the LIG samples at different magnifications. Functionalization with urea led to significant morphological changes, as evidenced by an increase in pore size at higher magnification (Figure 2d–f). The thermal decomposition of urea during laser irradiation released gases that generated internal pressure and promoted structural exfoliation, resulting in larger pores.^30,31^ SEM analysis of the LIG/Ni­(NO_3_)_2_ + Urea sample (Figure 2g–i)), conducted at different magnification levels, revealed a uniform distribution of microparticles within the carbon matrix. These nickel-derived particles embedded in the LIG matrix may act as electrical current amplifiers in the electrochemical sensor, thereby enhancing electrocatalytic activity.^32,33^

**Figure 2:**
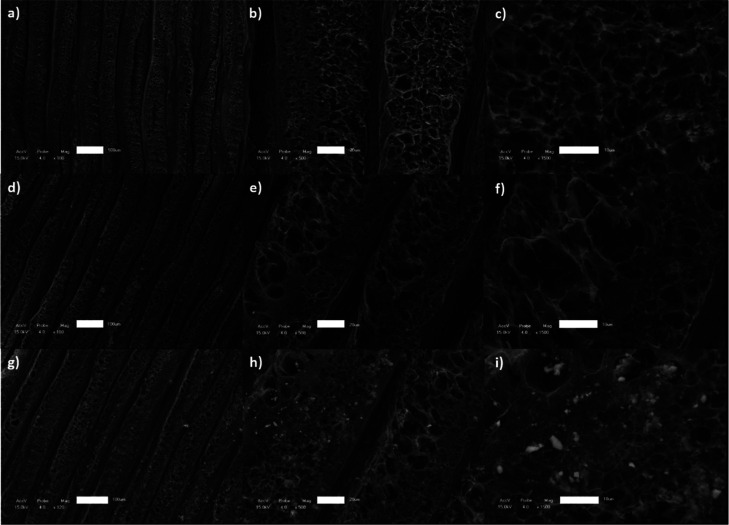
Comparative SEM micrographs of (a–c) LIG, (d–f) LIG/Urea, and (g–i) LIG/Ni­(NO_3_)_2_ + Urea composites at different magnification levels.

To verify the presence of nitrogen functionalization, CHN elemental analysis was conducted to quantify nitrogen-containing groups in four different material systems: pristine LIG, LIG/Urea, LIG/Ni­(NO_3_)_2_, and LIG/Ni­(NO_3_)_2_ + Urea. As shown in Table S1, the LIG/Ni­(NO_3_)_2_ + urea composite exhibited a 13.08% increase in nitrogen content compared to the corresponding formulation without urea. This result confirms the successful incorporation of nitrogen into the carbon-based matrix of the LIG. As reported before, nitrogen-rich functional groups may enhance electrical signal amplification, highlighting their importance in improving the electrochemical performance of the sensor.^34^

In the Raman spectra of LIG, the D, G, and 2D bands are key indicators of the material’s structure and quality. The Raman spectroscopy of the samples is shown in Figure 3. The G band is associated with the in-plane stretching of sp^2^ carbon atoms, and it provides information about the ordering within the graphitic structure. The D band reflects structural disorder and defects in the carbon lattice, such as vacancies, sp^3^ hybridization, or edge effects, and becomes active in the presence of imperfections, i.e., higher intensity in this band indicates a greater level of disorder. The 2D band is a second-order overtone of the D band and is related to the stacking order and number of graphene layers.^31,35^ The analysis of the relative intensities of these bands, specifically the *I*_D_/*I*_G_ and *I*_2D_/*I*_G_ ratios, is essential to evaluate the degree of graphitization and defect density in LIG. As illustrated in Figure 3, nitrogen incorporation via urea doping increases the I_D_/I_G_ ratio, reflecting greater structural disorder and defect density in the graphene lattice. This behavior is typical of oxidation processes or heteroatom introduction, as supported by the CHN elemental analysis. The increase in the *I*_2D_/*I*_G_ ratio observed after doping suggests a decrease in the number of graphene layers. Therefore, the simultaneous increase in the I_D_/I_G_ ratio suggests that nitrogen doping reduces the number of graphene layers while increasing defect density.^36,37^

**Figure 3:**
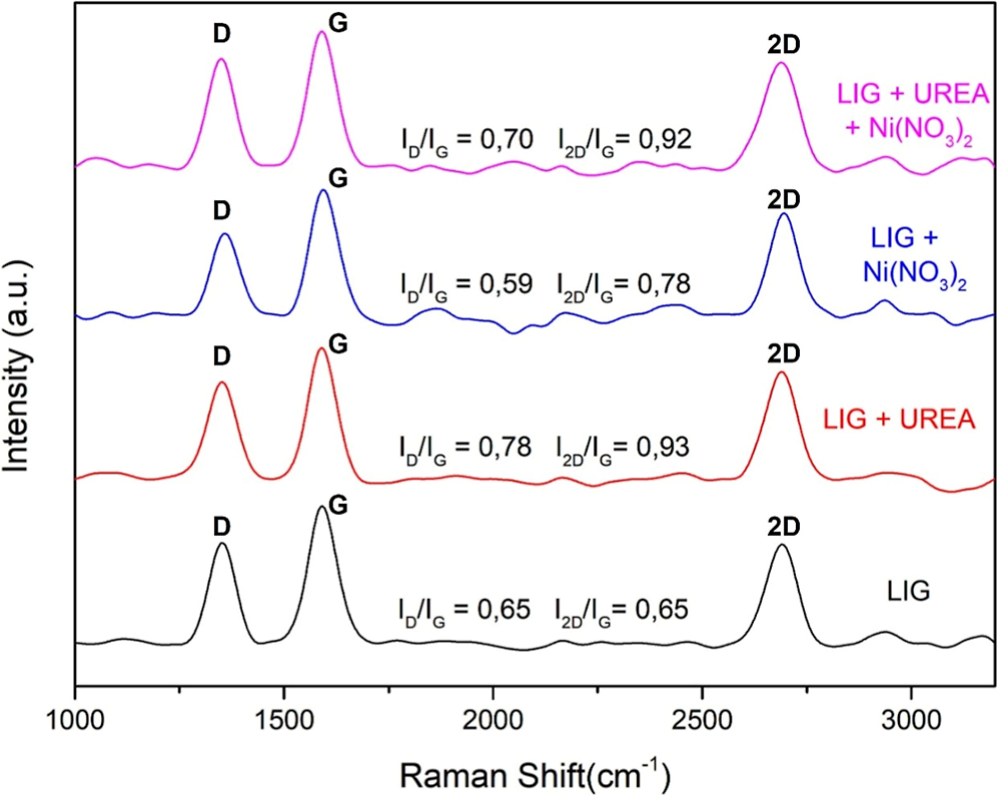
Raman spectra of LIG, LIG/Urea, LIG/Ni­(NO_3_)_2_, and LIG/Ni­(NO_3_)_2_ + Urea electrodes.

To reinforce the results and the process of nitrogen incorporation, FTIR analysis (Figure S1) was performed to understand, which functional groups are present on the sensor’s surface. The FTIR analysis of the LIG/Ni­(NO_3_)_2_ + urea sensor confirms the successful functionalization of the LIG matrix. A broad band at 3594 cm^–1^ is attributed to the O–H and N–H stretching vibrations,^38^ originating from both residual moisture and the urea precursor. The characteristic Amide I band, dominated by the C=O stretching vibration, is observed at 1654 cm^–1^, while the Amide II band, primarily resulting from N–H in-plane bending, appears at 1541 cm^–1^.^39^ The presence of both bands provides strong evidence of effective urea integration. Furthermore, the peak at 1504 cm^–1^ corresponds to the C=C stretching of the LIG aromatic skeleton, while the signal at 1702 cm^–1^ suggests C=O stretching from residual oxidation during the laser induction process.^40^ Regarding the nitrogenous and metallic species, the peaks at 1459 cm^–1^ (C–N stretching), 1397 cm^–1^ (asymmetric NO_3_^–^ stretching), and 670 cm^–1^ (Ni–O/Ni–OH lattice vibrations) confirm the successful incorporation of nickel nitrate and urea into the sensor’s active surface.^41–43^

### 3.2 Electrochemical Results

To investigate the electrochemical detection capability of the LIG/Ni­(NO_3_)_2_ + Urea electrode, CV measurements were carried out in the absence and presence of DA, and the results are shown in Figure 4a. As reported in the literature, sensors based on carbonaceous materials exhibit high reactivity toward DA oxidation.^32,44^ The superior electrocatalytic performance of the LIG electrode modified with nickel nitrate and urea is attributed to the synergistic effect between the nickel nanoparticles and nitrogen doping within the graphene matrix. During the laser writing process, the thermal decomposition of urea not only promotes the formation of a highly porous graphene structure, with an increased effective surface area, but also facilitates the incorporation of nitrogen active sites, which modulate the local electronic density and favor the adsorption of dopamine molecules.^32,45^

**Figure 4:**
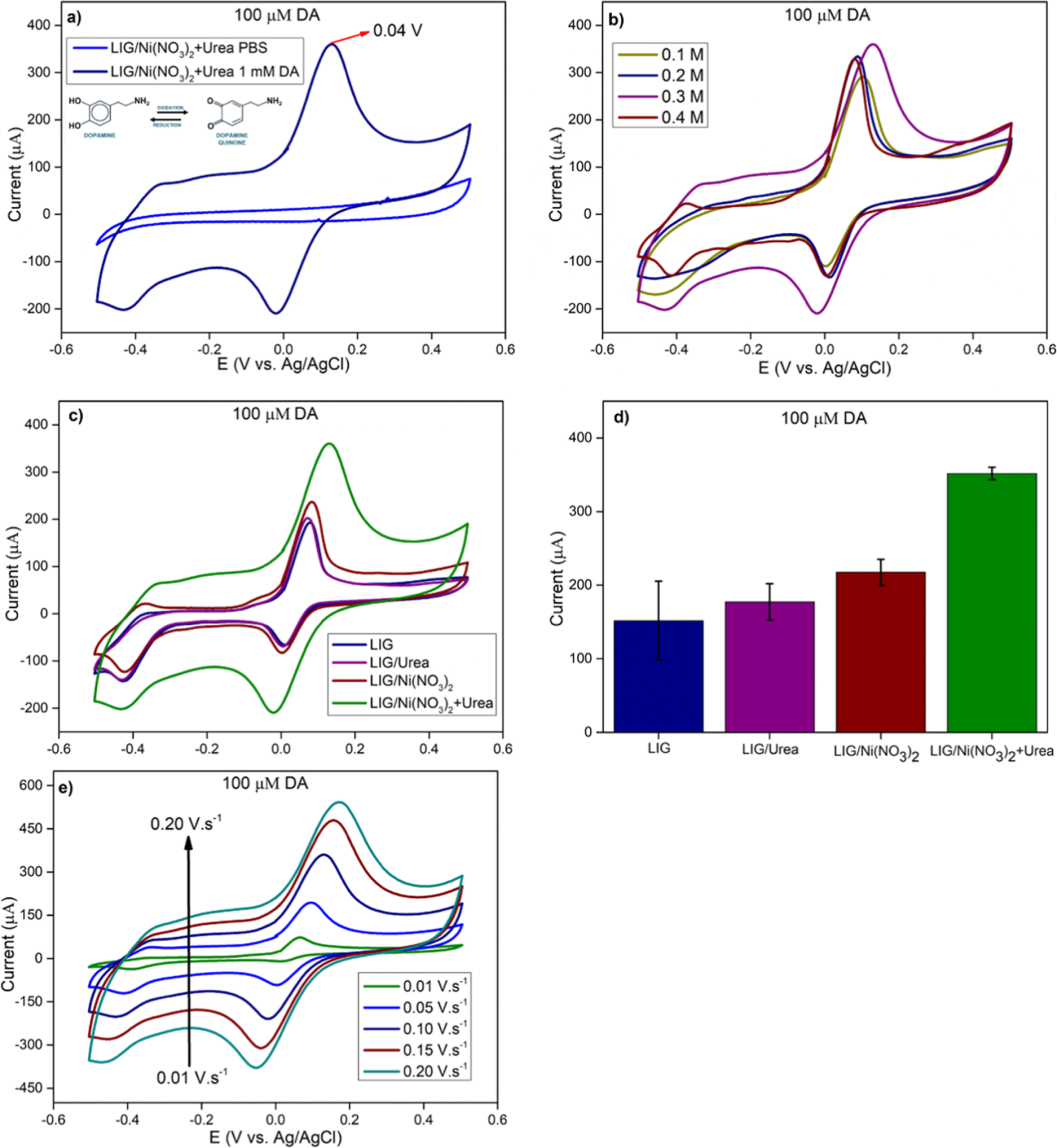
(a) CV of the LIG/Ni­(NO_3_)_2_+ Urea sensor in PBS and 100 μmol·L^–1^ DA. Schematic representation of dopamine reversible redox to dopamine-quinone under electrochemical conditions, pH = 7.5. (b) Evaluation and performance of different urea concentrations 0.1 to 0.4 mol·L^–1^in the sensor dispersion to optimize electrical current response. (c) CV comparative of the LIG sensor, LIG/Urea, LIG/Ni­(NO_3_)_2_, and LIG/Ni­(NO_3_)_2_ + Urea in the presence of 100 μmol·L^–1^DA. (d) Performance of LIG, LIG/Urea, LIG/Ni­(NO_3_)_2_, and LIG/Ni­(NO_3_)_2_ + Urea sensors for the detection of 100 μmol·L^–1^ DA. (e) CV performed at different scan rates using the LIG/Ni­(NO_3_)_2_+ Urea sensor for 100 μmol·L^–1^DA in PBS at pH = 7.5, following the equation *I*_p_ = (2.69 × 10^5^) *n* ^3/2^AD^1/2^C^0^v^1/2^.

The oxidation mechanism of dopamine involves the transfer of two electrons and two protons to form dopamine quinone (DAQ), a process catalyzed by the presence of nickel species (such as NiO or Ni­(OH)_2_/NiOOH), which act as redox mediators and signal amplifiers.^46,47^ Simulations based on density functional theory in analogous systems indicate that the interaction between the nickel metal centers and the nitrogen-functionalized groups reduces the activation energy for interfacial charge transfer, resulting in more defined oxidation peaks and significantly higher peak currents compared to unmodified LIG.^32,48^ This hybrid architecture not only enhances the electrical conductivity of the sensor but also stabilizes the catalytic sites, ensuring high sensitivity and selectivity even in complex matrixes, such as synthetic tear fluid. A well-defined oxidation peak was observed at 0.04 V, demonstrating that the electrode exhibits a sensing capability for DA oxidation.

At this potential, the catechol group in the dopamine molecule undergoes oxidation, forming dopamine-o-quinone through the loss of two electrons and two protons. This redox transformation involves the formation of covalent bonds between the aromatic ring and adjacent oxygen atoms, as illustrated in the inset of Figure 4a. In the subsequent reduction step, observed near −0.1 V, the quinone structure is reduced back to dopamine, completing the reversible redox cycle.^49^

To investigate and determine the optimal urea concentration for DA detection, CV measurements were performed using LIG/Ni­(NO_3_)_2_ electrodes and urea concentrations ranging from 0.1 to 0.4 mol·L^–1^ (Figure 4b). The tests were conducted in a 100 μmol·L^–1^ DA solution, using PBS as the supporting electrolyte. The inset displays the values of the current response for each sensor. The highest current response was observed for the sensor functionalized with 0.3 mol·L^–1^ urea. These results can be attributed to the synergistic effect between the nickel-based particles and the nitrogen doping introduced by urea functionalization. As demonstrated in the SEM, urea enhances the porosity of the LIG structure, while the dispersed nickel-based particles can act as selective redox centers for DA, promoting interactions with the nitrogen-containing groups.^30,44,50^ The incorporation of nitrogen into the electrochemical sensor modulates its electronic properties by introducing active sites, which improve conductivity and electrocatalytic activity toward dopamine oxidation, resulting in enhanced sensitivity and selectivity.^51,52^ Moreover, urea’s biocompatibility supports its application in biological fluids such as blood, urine, and tears, expanding the sensor’s potential for use in noninvasive systems.^53^

To further confirm the best-performing sample, LIG-based electrodes functionalized in different ways were tested using CV, with 100 μmol·L^–1^ DA as the analyte and PBS as the supporting electrolyte. CV curves in the absence and presence of 100 μmol·L^–1^ DA for LIG, LIG/Urea, and LIG/Ni­(NO_3_)_2_ are shown in Figures S2, S3, and S4, respectively. Figure 4c shows the comparison of CV curves for unmodified LIG, LIG/Urea, LIG/Ni­(NO_3_)_2_, and LIG/Ni­(NO_3_)_2_ + Urea.

The redox processes involving nickel in alkaline environments can lead to interconversion between different nickel oxidation states, primarily Ni­(II) and Ni­(III). These processes typically involve the formation of nickel hydroxides and oxyhydroxides on the electrode surface. The appearance of a shoulder at 0.2 V in the voltammetry of the LIG/Ni­(NO_3_)_2_ sensor suggests an electrochemical process occurring at a very close potential. This feature could be attributed to a Ni­(II)/Ni­(III) redox transition at a specific nickel site or phase, which occurs at a potential slightly different from that of the main peak.^54^ An alternative hypothesis is that a shoulder may appear if charge transfer is limited or if the process is diffusion-controlled within a certain potential range (the DA oxidation process), while another process with different kinetics (the Ni­(II)/Ni­(III) redox transition) occurs in parallel. The morphology of the electrode surface and the way nickel is deposited or incorporated can create different electrochemical environments, leading to variations in peak potentials.^55–57^

The LIG/Ni­(NO_3_)_2_ + Urea sensor exhibits more pronounced and sharper redox peaks in the presence of DA, indicating significantly improved electrochemical performance. Thus, a significantly higher oxidation current was observed, as shown in Figure 4d. This increase can be attributed to the electrocatalytic properties of the modifiers, which facilitate electron transfer and amplify the sensor’s signal, increasing its maximum current response and thereby expanding the detectable concentration range of DA.^58^ The incorporation of nickel species plays a key role in facilitating redox mediation and improving the system’s electron transfer kinetics. Additionally, the nitrogen functionalities introduced through urea doping enhance electrical conductivity and create additional active sites for DA adsorption. These synergistic effects result in reduced overpotential and increased peak current, leading to improved sensitivity and selectivity for DA detection.^59^

Figure 4e shows the behavior of the DA sensor at varying scan rates (from 0.01 V·s^–1^ to 0.20 V·s^–1^), where the peak current increases linearly with the square root of the scan rate, which is characteristic of a diffusion-controlled process.^53–55^ This indicates that the oxidation kinetics of DA on the sensor are governed by the diffusion of the analyte to the electrode surface. Furthermore, the electrochemical reaction is fast and reversible. Increasing the current range at higher scan rates enhances the sensor’s dynamic sensitivity, enabling the detection of DA at lower concentrations by more efficiently renewing the diffusion layer.^56^ The oxidation and reduction peak potentials are seen to be shifted toward more positive and negative areas, respectively, as the scan rate increases. This is consistent with a slower electron transfer mechanism at higher scan rates.^57^

To quantitatively substantiate the enhanced catalytic activity of the modified sensors, the electrochemical active surface area (ECSA) for all electrode compositions was determined by using the Randles–Sevcik equation. The calculated ECSA values were 3.27 cm^2^ for pristine LIG, 3.44 cm^2^ for LIG/Urea, 4.02 cm^2^ for LIG/Ni­(NO_3_)_2_, and a remarkable 13.36 cm^2^ for the LIG/Ni­(NO_3_)_2_ + Urea composite. The isolated addition of urea or nickel species resulted in only modest increases in the electroactive area. In stark contrast, the synergistic comodification promoted an approximately 4-fold enhancement in ECSA compared to the pristine material. This quantitative finding strongly corroborates the morphological evidence (Figure 2), demonstrating that the simultaneous thermal decomposition of urea and incorporation of nickel during laser induction creates a highly exfoliated, 3D porous network that maximizes the exposed active sites, thereby facilitating superior dopamine electrooxidation kinetics.

Tests were carried out to evaluate the performance of the LIG/Ni­(NO_3_)_2_ + Urea sensors in terms of repeatability, reproducibility, and stability for the detection of dopamine in tear samples. The repeatability test (Figure S5) was performed with five successive measurements (*n* = 5). The relative standard deviation (RSD) was calculated, resulting in an RSD of 1.29%, which indicates that the LIG/Ni­(NO_3_)_2_ + Urea electrochemical sensors exhibit a high repeatability. Reproducibility tests (Figure S6) were conducted using three different sensors in triplicate, yielding an RSD of 3.17%, confirming that the fabrication process is highly reproducible. The stability of the disposable sensor (Figure S7) was monitored over a 30-day period (1, 7, and 30 days). The results demonstrated that the sensor remains fully operational for point-of-care testing, maintaining an ideal performance for up to 7 days, with a gradual decrease in response observed by day 30.

Figure 5a displays the results of DPV for the LIG/Ni­(NO_3_)_2_ + Urea sensor at various DA from 0.25 to 16.44 μmol·L^–1^. DPV was selected due to its enhanced sensitivity compared to CV, as it minimizes capacitive current interference. Initial measurements were performed in PBS at pH 7.5 to establish a baseline, followed by incremental additions of DA. As the DA concentration increased, the oxidation current rose correspondingly. As depicted in Figure 5b, the current response showed a linear correlation (*R* ^2^ = 0,98) with DA concentration within the tested range. Using the calibration curve, the limit of detection (LOD) and limit of quantification (LOQ) are calculated according to eqs 1 and 2,^60^ where *S* is the standard deviation of the PBS samples.

1 $$LOD=\frac{\left(3.3^{*}S\right)}{\left|Slope\right|}$$

2 $$LOQ=\frac{\left(10^{*}S\right)}{\left|Slope\right|}$$

**Figure 5:**
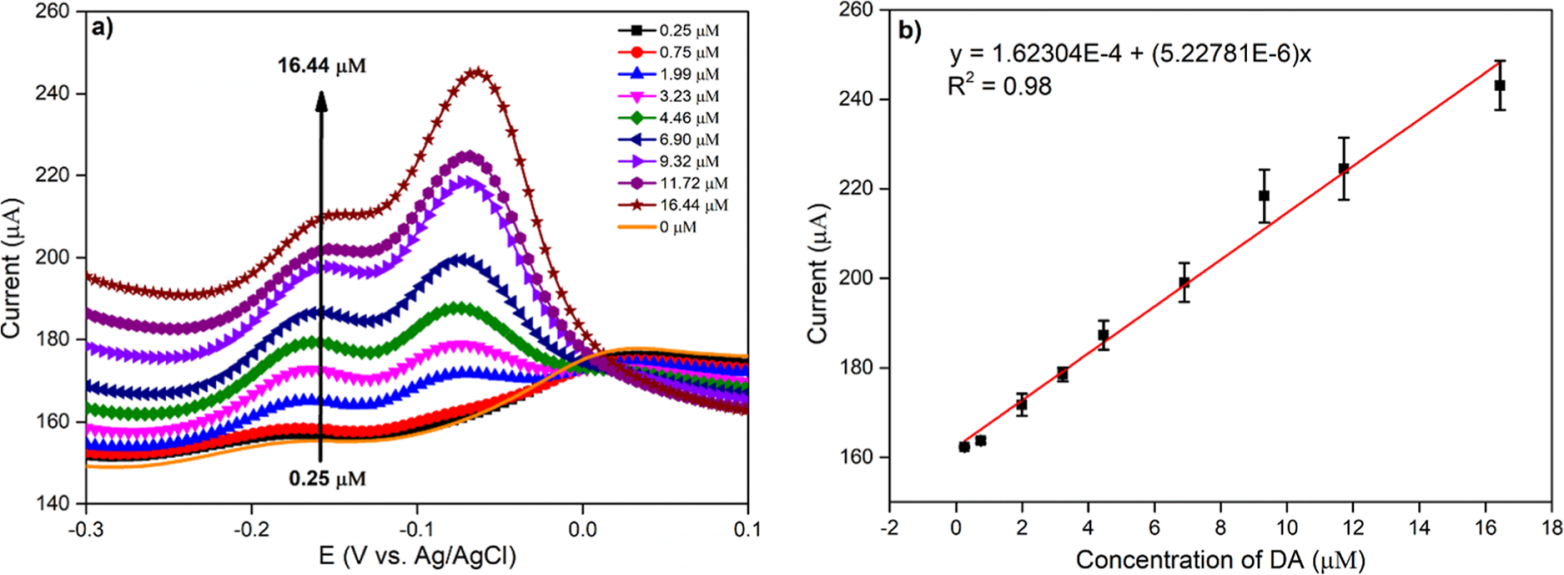
(a) DPV employed to evaluate the sensor’s analytical performance for DA detection inserted in PBS solution. (b) Analytical curve of the sensor in PBS solution.

Based on the calibration data, the LOD and LOQ were 17.86 nmol·L^–1^ and 54.14 nmol·L^–1^, respectively. The DPV results shown in Figure 5b revealed a highly linear response (*R* ^2^ = 0.98) across the tested dopamine concentration range. These performance metrics highlight the sensor’s remarkable sensitivity and are consistent with those of other sensors reported in the literature, as shown in the comparative Table 1.

**Table 1:** Comparison of Performance of Sensors from Other Authors with This Work

electrode	analyte	techniques	linear range (DA)	LOD (DA)	refs
rGO/ReO_3_ nanocomposite-modified GCE	DA/UA/AA	DPV	10–152 nM	0.08 nM	^61^
Nb_4_C_3_T_*x*_ MXene-AgNPs LIG	DA	CA	1.1–10 μM	1.0 nM	^62^
MoS_2_/Au	DA	DPV	0.5–300 μM	76 nM	^63^
MoS_2_-RGO	DA/UA/AD	DPV	MoS_2_-RGO: 1–110 μM	MoS_2_-RGO: 0.5 μM	^64^
BC_5_N			BC_5_N: 2.3–20 μM	BC_5_N:2.1 μM	
rGO-SS	DA	DPV	1–1000 μM	1 μM	^65^
LIG/Ni(NO_3_)_2_ + urea	DA	DPV	0.25–16.44 μM	17.86 nM	This Work

## 4 Real Sample Analysis

Electrochemical detection of DA in biological fluids, such as tear fluid, represents a promising approach for noninvasive monitoring of neurological and metabolic disorders. Recovery experiments were performed by spiking the samples with the DA at four different concentration levels (3.23–9.32 μmol·L^–1^). All measurements were carried out in triplicate (n = 3), and concentrations were calculated using the external standard method. The recovery values ranged from 99.7% to 100.1% (Table 2), with low RSD values, indicating the high accuracy and precision of the proposed sensor for DA determination in real matrices.

**Table 2:** Application of LIG/Ni­(NO_3_)_2_ + Urea Sensor for Determination DA in Synthetic Tear

sample	added DA (mol·L^–1^)	found DA (mol·L^–1^)^a^	recovery (sensor, %)^b^
synthetic tear	(3.23 × 10^–6^)	(3.23 ± 0.0103) × 10^–6^	100
	(4.46 × 10^–6^)	(4.46 ± 0.0100) × 10^–6^	100
	(6.90 × 10^–6^)	(6.88 ± 0.0085) × 10^–6^	99.7
	(9.32 × 10^–6^)	(9.33 ± 0.0087) × 10^–6^	100.1

a Average of 3 measured concentrations.

b $Recovery\;percentage=\left(\frac{\left(Found\right)}{Added}\right)\cdot 100$

The electrochemical performance in synthetic tear fluid was evaluated within a concentration range relevant to clinical monitoring (3.23–9.32 μmol·L^–1^). In this low-concentration regime, the sensor maintained a stable and linear increase in oxidation current (*R* ^2^ = 0.96), demonstrating that matrix-induced fouling is negligible under these conditions. However, it is important to note that the fouling phenomenon, characterized by the formation of an insulating polydopamine film, is a concentration-dependent process typically observed at much higher DA levels.^66–68^ At the lower concentrations tested in this study, the porous LIG/Ni­(NO_3_)_2_ + Urea architecture effectively mitigates surface passivation, ensuring that the analytical signal remains proportional to the analyte concentration. This confirms the sensor’s reliability for dopamine detection in real-world tear samples without the interference of significant electrode fouling.

Furthermore, the error bars showed variations between 2% and 6%, indicating the precision and reliability of the proposed sensor. However, as shown in Figure S8, small variations in current during DA detection were observed, attributed to the presence of interferents in the synthetic tear fluid. Each interfering was analyzed individually to assess its impact on sensor performance.

## 5 Conclusion

This study reported the development of electrochemical sensors based on LIG for the detection of dopamine in both controlled environments and synthetic tear fluid. Optimization studies revealed that the functionalization of the working electrodes with 0.1 mol·L^–1^ Ni­(NO_3_)_2_ and 0.3 mol·L^–1^ urea resulted in an improved performance for nonenzymatic electrochemical sensing of DA. Surface morphology analysis revealed significant differences between treated and untreated electrode, resulting in increased electroactive surface area and enhanced sensor performance. The sensor exhibited well-defined detection limits and a clear dynamic range for dopamine quantification. Notably, the incorporation of urea into the LIG/Ni­(NO_3_)_2_ system increased the oxidation current in the presence of dopamine while maintaining high sensitivity, thereby improving selectivity and detection performance. In PBS solution, the sensor had a response in the detection range of 0.25–16.44 μmol·L^–1^ LOD of 17.86 nmol·L^–1^ and LOQ of 54.14 nmol·L^–1^, with *R* ^2^ = 0.98. In synthetic tear fluid, the sensor maintained a reliable response with a linear range of 3.23–9.32 μmol·L^–1^, with recovery for DA close to 100%. Notably, the direct laser fabrication of nickel-based microstructures represents a novel, cost-effective, and efficient strategy. The LIG/Ni­(NO_3_)_2_ + Urea sensor demonstrated strong potential for nonenzymatic DA detection in both standard and biologically relevant samples, and the innovative laser synthesis method introduced here offers a promising path forward in electrochemical sensing.

## Supplementary Material



## Notes

CHN elemental analysis data for nitrogen quantification across the different sensor configurations; FTIR spectrum of the LIG/Ni­(NO_3_)_2_ sensor; cyclic voltammetry characterization of the pristine LIG, LIG/Urea, and LIG/Ni­(NO_3_)_2_ sensors in the presence of dopamine; repeatability, reproducibility, and storage stability evaluations of the LIG/Ni­(NO_3_)_2_ + Urea sensor configuration; and selectivity testing against potential interfering components in synthetic tears This material is available free of charge via the Internet at https://pubs.acs.org/journal/acsodf (PDF)
