# Supplementary material for: Toward Non-Invasive Neurological Biomarker Monitoring: Dopamine Sensing in Tears with Laser-Induced Graphene Electrochemical Sensors
Source: ACS Omega. 2026 Jun 9;11(24):36141–50. doi: 10.1021/acsomega.6c03287 (PMC13294949; doi:10.1021/acsomega.6c03287)
Supplement: Supplementary file 1 [file ao6c03287_si_001.pdf]

# Towards Non-Invasive Neurological Biomarker Monitoring: Dopamine Sensing in Tears with Laser induced graphene electrochemical sensors

Lucas Minghini Gonçalves[a,b], Bruno Vasconcellos Lopes[a,b], Bruno da Silveira Noremberg[a], Raphael Dorneles Caldeira Balboni[a], Guilherme Kurz Maron[a,c], Anderson Thesing[d], Daiane Dias[e], Irene Teresinha Santos Garcia[f], Sabir Khan[a], Neftali Lenin Villarreal Carreno[a,b]*

[a] *Graduate Program in Materials Science and Engineering, Technology Development Center, Federal University of Pelotas, 96010-000 Pelotas, Rio Grande do Sul, Brazil*

[b] *Center for Embedded Devices and Research in Digital Agriculture (CEDRA), São Leopoldo, RS, 93025-753, Brazil*

[c] *Northern Regional Technological Institute (ITR Norte), Technological University of Uruguay (UTEC), 40000, Rivera/Rivera, Uruguay*

[d] *Institute of Physics, Universidade Federal do Rio Grande do Sul, Porto Alegre RS, 91501-970, Brazil*

[e] *Universidade Federal do Rio Grande (FURG) – School of Chemistry and Food, Av. Itália S/N, km. 8 (Carreiros), Rio Grande, RS, 96203-900, Brazil*

[f] *Federal University of Rio Grande do Sul, Department of Physical Chemistry, , Porto Alegre, RS, 91501-970, Brazil*

**Corresponding author: N.L.V. Carreno* (e-mail: *neftali@ufpel.edu.br).*

## RESULTS

**Table S1.** CHN elemental analysis was conducted to quantify the nitrogen (N) content across the four sensor configurations.

| Sample | Nitrogen |
|---|---|
| LIG | 2.05% |
| LIG + urea | 39.39% |
| LIG + $Ni(NO_3)_2$ | 6.34% |
| LIG + urea +$Ni(NO_3)_2$ | 19.42% |

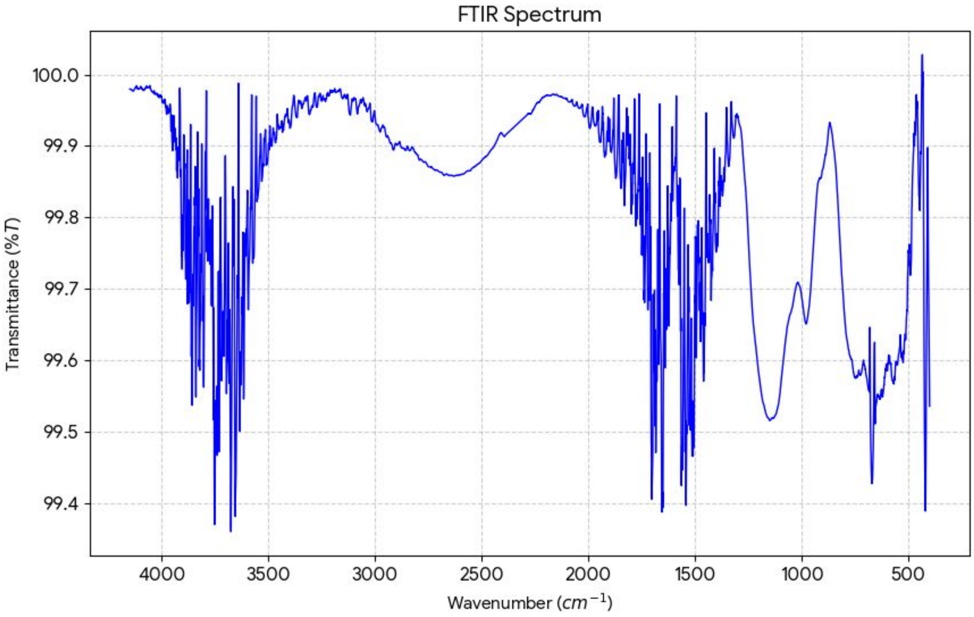


**Fig. S1.** FTIR of the LIG/Ni($NO_3$)$_2$ sensor.

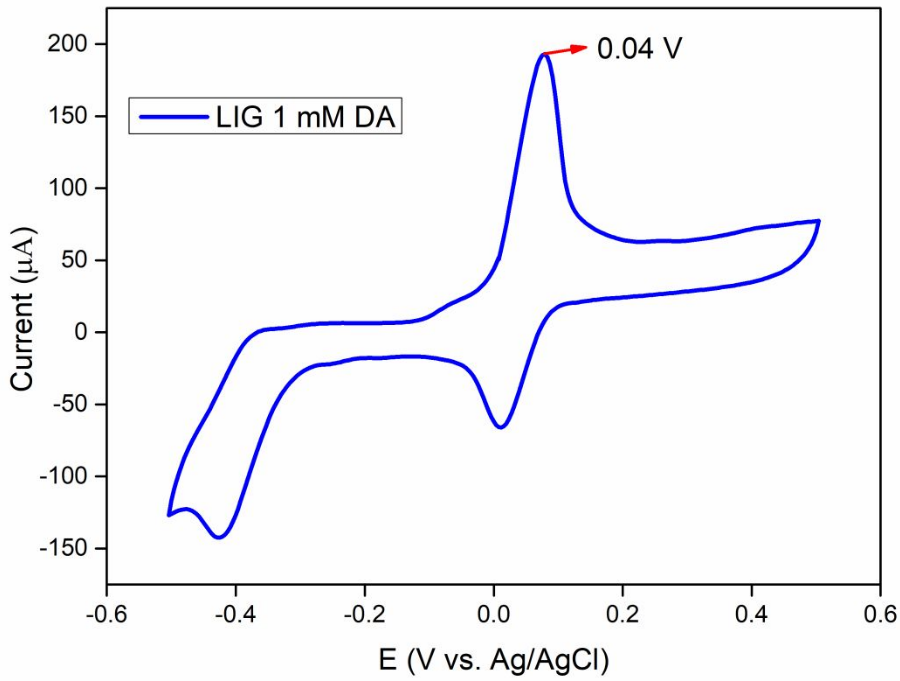


**Fig. S2.** CV of the LIG pristine sensor 1mM DA in PBS.

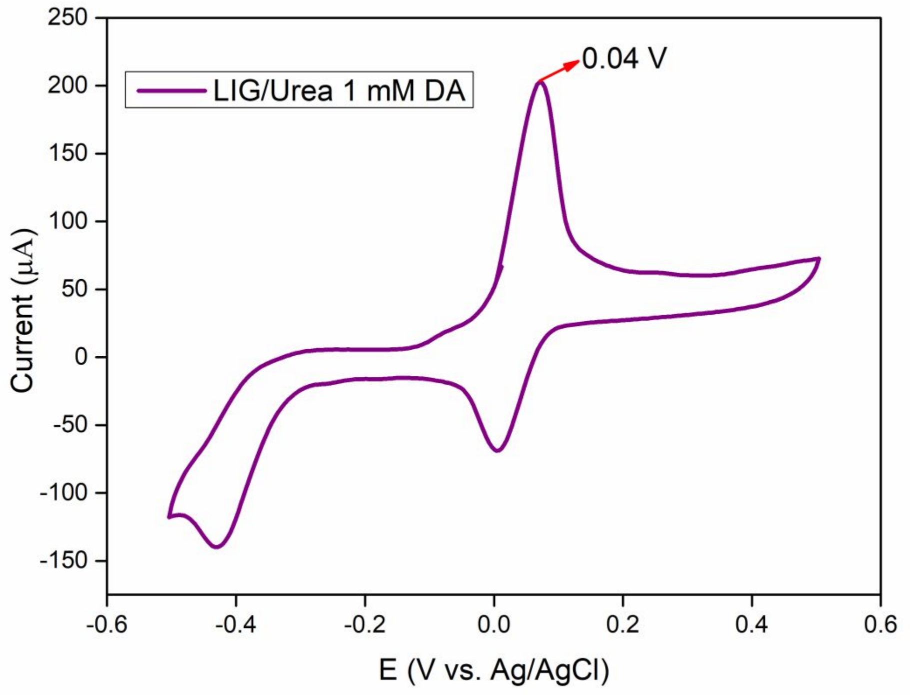


***Fig. S3.*** *CV of the LIG/Urea sensor 1mM DA in PBS.*

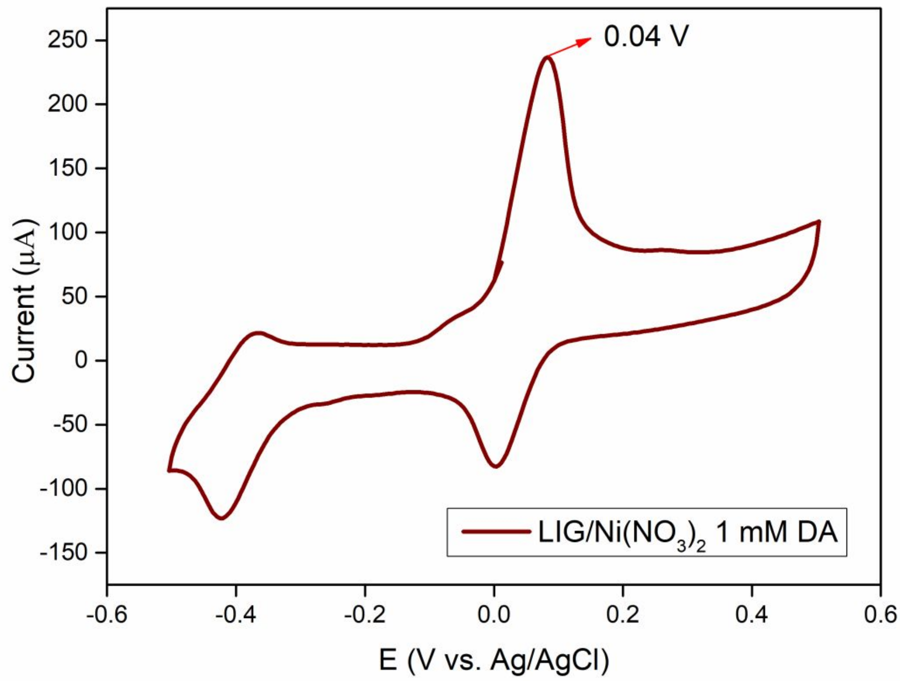


**Fig. S4.** CV of the LIG/$Ni(NO_3)_2$ sensor 1mM DA in PBS.

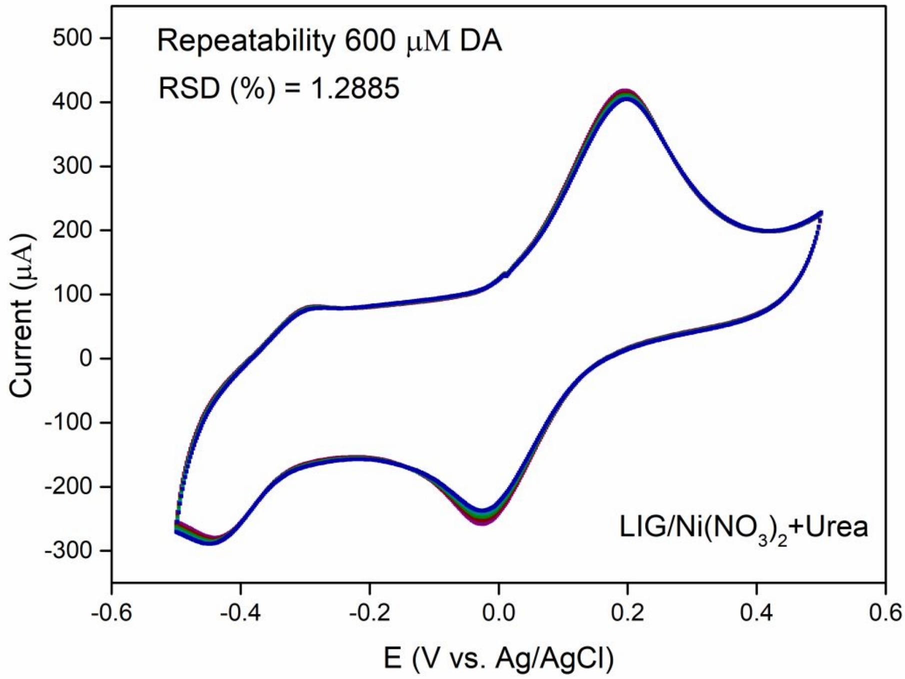


**Fig. S5.** Repeatability LIG/Ni$(NO_3)_2$ + urea for 600 μmol $L^{-1}$ with RSD (%) = 1.2885.

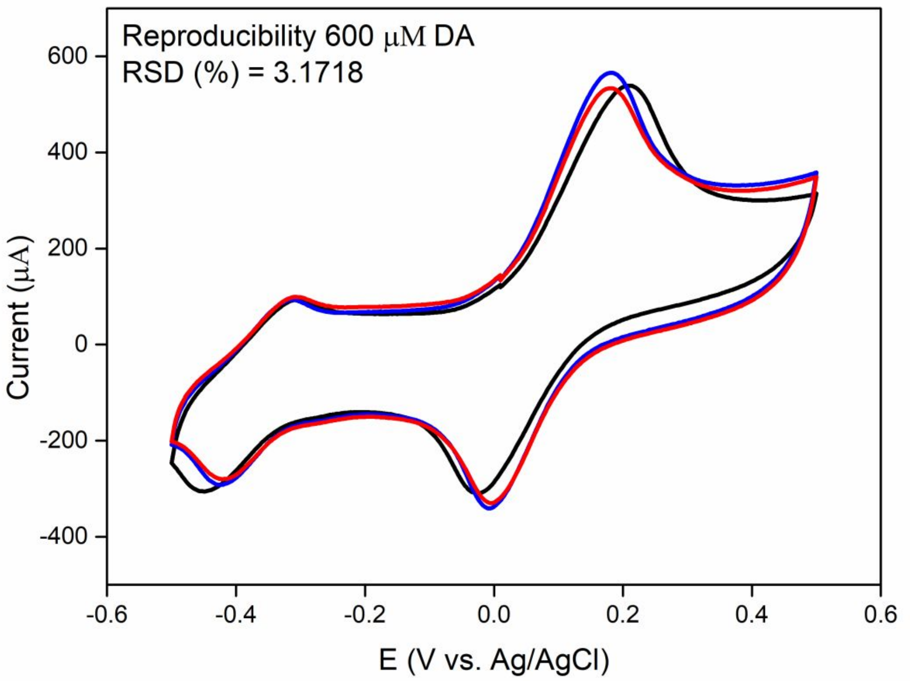


**Fig S6.** Reproducibility for three different sensors for CV analyses of the LIG/$Ni(NO_3)_2$ + urea for 600 μmol $L^{-1}$ with RSD (%) = 3.1718.

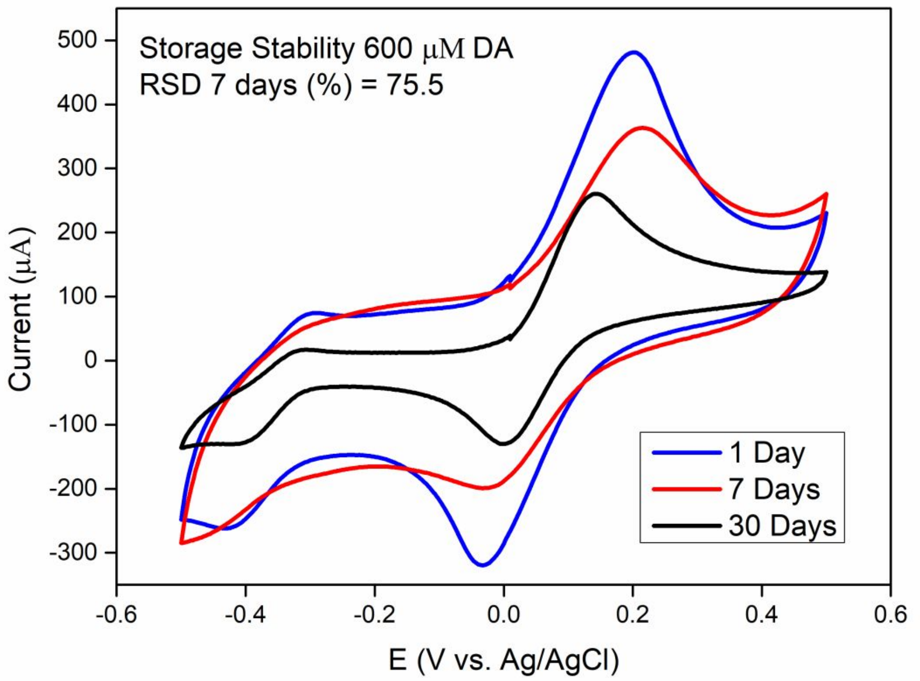


***Fig S7.*** Storage stability the sensor for CV analyses of the LIG/Ni$(NO_3)_2$ + urea in different period 1,7 and 30 days and RSD (%) for 7 days = 75.5.

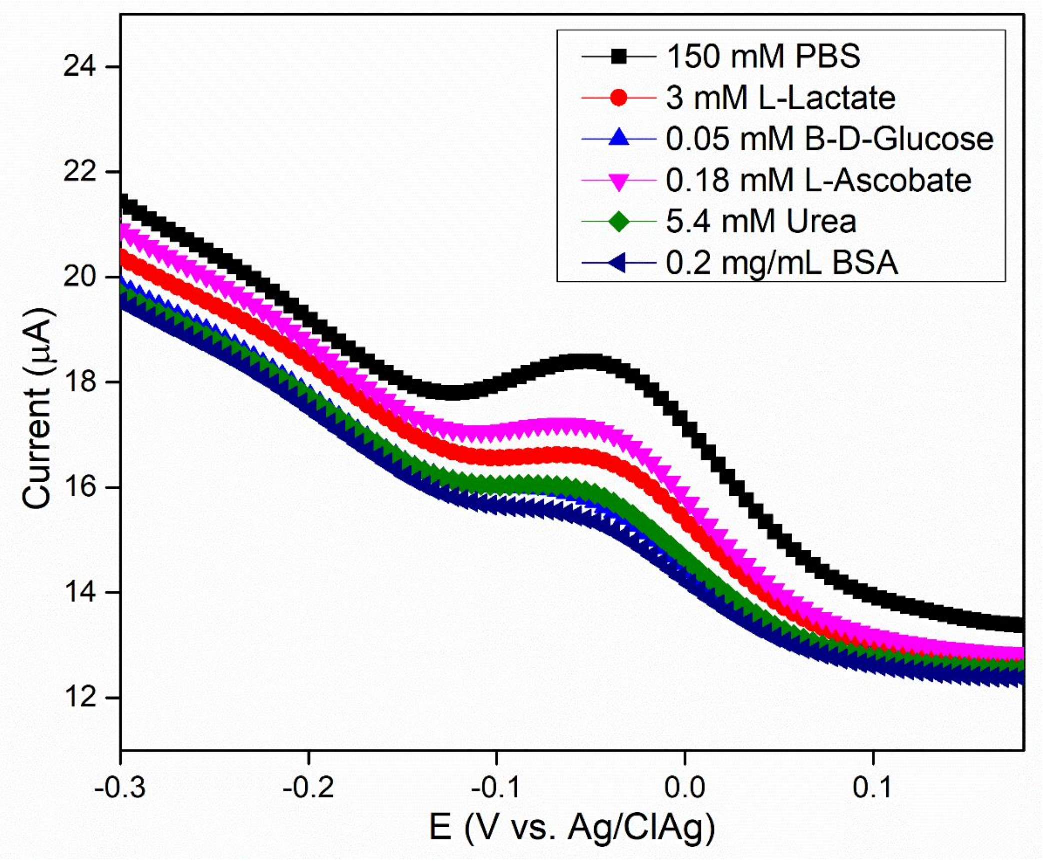


**Fig. S8.** Different compositions present in the synthetic tear tested individually on the sensor as possible interferences for the sensor, 150 mM PBS, 3 mM L-Lactate, 0.05 mM β-D-Glucose, 0.18 mM L-Ascorbate, 5.4 mM Urea, 0.2 mg/mL BSA.
